# In search of the moral-psychological and neuroevolutionary basis of political partisanship

**DOI:** 10.1590/1980-57642016dn11-010004

**Published:** 2017

**Authors:** Vitor Geraldi Haase, Isabella Starling-Alves

**Affiliations:** 1 Departamento de Psicologia, Universidade Federal de Minas Gerais, Belo Horizonte MG, Brazil.; 2 Programa de Pós Graduação em Neurociências, Universidade Federal de Minas Gerais, Belo Horizonte MG, Brazil.; 3 Programa de Pós-Graduação em Psicologia: Cognição e Comportamento, Universidade Federal de Minas Gerais, Belo Horizonte MG, Brazil.; 4 Instituto Nacional de Ciência e Tecnologia: Comportamento, Cognição e Ensino.

**Keywords:** personality, partisanship, politics, self-deception

## Abstract

In many countries, a radical political divide brings several socially relevant decisions to a standstill. Could cognitive, affective and social (CAS) neuroscience help better understand these questions? The present article reviews the moral-psychological and neuroevolutionary basis of the political partisanship divide. A non-systematic literature review and a conceptual analysis were conducted. Three main points are identified and discussed:

1) Political partisan behavior rests upon deep moral emotions. It is automatically processed and impervious to contradiction. The moral motifs characterizing political partisanship are epigenetically set across different cultures;
2) partisanship is linked to personality traits, whose neural foundations are associated with moral feelings and judgement;
3) Self-deception is a major characteristic of political partisanship that probably evolved as an evolutionary adaptive strategy to deal with the intragroup-extragroup dynamics of human evolution. CAS neuroscience evidence may not resolve the political divide, but can contribute to a better understanding of its biological foundations.

## Abstract (pt)

Em diversos países, uma divisão política radical leva à estagnação de várias decisões socialmente relevantes. Poderia a neurociência cognitiva, afetiva e social (CAS) contribuir para a compreensão dessas questões? O presente artigo revisa as bases morais psicológicas e neuroevolutivas do partidarismo político. Uma revisão não-sistemática da literatura e uma análise conceitual foram realizadas. Três pontos principais são identificados e discutidos:

1) o comportamento político partidário tem como base emoções morais profundas. Ele é automaticamente processado e insensível à contradição. Os motivos morais que caracterizam o partidarismo político são definidos epigeneticamente através de diferentes culturas;
2) partidarismo político está ligado a traços de personalidade, cujas fundações neurais estão associadas a sentimentos e julgamentos morais;
3) O auto-engano é uma das principais características de partidarismo político, que provavelmente evoluiu como estratégia evolutiva adaptativa para lidar com a dinâmica intragrupo-extragrupo da evolução humana. As evidências da neurociência CAS podem não resolver a divisão política, mas podem contribuir para uma melhor compreensão de seus fundamentos biológicos.

## INTRODUCTION

Developing countries, including Brazil, are plagued by numerous socioeconomic problems such as corruption, lack of judicial stability, socioeconomic inequality, poor education, etc. It is a debatable political question whether this situation is improvable by any "rational" policies and which means of improvement are recommended, if at all. On the one hand, efforts towards rationally planned social betterment are to be avoided at any rate according to conservative political positions.^1^ On the other hand, liberals prescribe rationally planned distribution of wealth as a means of eradicating inequality.^2^ These are political questions that must be elucidated before any contribution of cognitive, affective and social (CAS) neuroscience to develop society can be possible. Settling these political questions is obviously no trivial task. A mission impossible, it could be said. Clearly, CAS neuroscience could help to elucidate the neuroevolutionary underpinnings of the political controversies underlying social development.

This brings us to a second point: is CAS neuroscience mature enough to contribute to social human affairs in the sense of a paradigm shift? The answer seems to be no. And this is fortunate. CAS is not developed enough to generate its own hypotheses nor a new epistemology that would, eventually, revolutionize the study of human social affairs. So far, CAS neuroscience, most notably functional neuroimaging, has served as a methodological device to test hypotheses developed in other fields, such as evolutionary theory, psychology, sociology, or economics. This situation is fortunate in the sense that CAS neuroscientists have much work ahead, before their research can more directly address social affairs. This point will be illustrated by discussing the moral psychological underpinnings of contemporary political dissent.

The political divide is self-evident in a number of countries across the Americas and elsewhere.^3,4^ Debate on the internet-based social networks is so heated to the point that long-term friendships may even be damaged on political grounds. Tempers are fierce regarding politics and the internet provides a ready, quick, cheap and dirty way of expressing them.

Taking the USA as an example, the political landscape of opinions may be summarized in the following way.^3^ Liberals (left partisans in the American sense of the word) adhere to an agenda of human rights, compensatory policies of state interventions in favor of equality, and political correctness regarding traditions (or habits). Conservatives plea for less state intervention, freedom of enterprise, consciousness and expression, traditional family and religious values, law and order, etc. Libertarians are situated in-between. While on the one hand, libertarians abhor any form of state intervention, on the other hand, they endorse a liberal agenda for moral affairs.

At first, the typology suggested by Haidt^3^ may seem oversimplified. But it is endorsed by empirical research on personality characteristics associated with each position.^5,6^ And it also has practical implications. In some countries, such as the USA and Brazil, public opinion is strictly divided into two halves between liberals and conservatives, with a tiny but growing number of libertarians in between.^3,4^ The political divide culminates in standstill regarding several matters of pressing social interest, such as social welfare, affirmative action, abortion, criminality, etc.

In this article, we set out to review and reflect on the possible moral-psychological and neuroevolutionary foundations of this political partisanship divide. We are interested in identifying studies that contribute to a better understanding of why people tend to fiercely align across political positions, and why it is so difficult to constructively work with politically different-minded individuals. We are also interested in identifying evidence regarding the possible neural underpinnings and evolutionary foundations of the political divide. We feel this is a necessary, albeit insufficient, condition to overcome the political divide.

## METHODS

In order to investigate the contributions of CAS neuroscience to political partisanship, a non-systematic literature review, followed by conceptual analysis, was conducted. Initially, the reference lists of the books by Haidt^3^ and Trivers^47^ were used as seeds to identify further literature concerning the neural underpinnings of political partisanship and self-deception. The more recent publications were identified through a PubMed search using the following terms (with respective yields): "haidt j AND politics" (5 articles), "trivers r AND self-deception" (3 articles), "personality AND partisanship" (5 articles), "politics AND fmri" (54 papers), "partisanship AND fmri" (1 paper), "self-deception AND fmri" (1 paper).

## RESULTS

Results of the conceptual analysis of the extant evidence will be discussed under the five respective headings: the moral-psychological underpinnings of political partisanship: the neural underpinnings of moral intuitionism; the cross-cultural set of moral motifs, personality and political partisanship; the evolutionary adaptive functions of political partisanship; and finally, self-deception serving to promote political partisanship.

**Moral-psychological underpinnings of political partisanship.** In very general terms, according to Berlin,^7^ it could be said that the political lines of division are polarized between two concepts of freedom. On the one side are those that defend a negative concept of liberty, in the sense of classical liberal economic theory.^8,9^ According to its negative concept, liberty should be understood as free entrepreneurship or freedom from state or any other kind of interference. According to this perspective, a free market is a precondition for wealth and reduction of poverty and, by extension, alleviating social inequality.

On the other side, Marxian and other authors inspired in the leftist tradition defend strong state interventions in the form of greater income and wealth taxations and redistributive policies.^2^ This point of view considers liberty in the positive sense, as characterized by Berlin.^7^ The concept of positive liberty promotes human rights, social justice and equality. According to this perspective, socioeconomic equality is a precondition for freedom.

It is easy to see that two radically distinct views of human nature, society and the economy hide amidst the political divide.^3,4^ It is not the business of neuroscience to adjudicate between these extremes. But CAS neuroscience could help to understand the deeper evolutionary and emotional origins of this political divide. This would be an important step forwards, helping to attenuate the political abyss and pave the way for more constructive and productive ways of cooperation between social forces.

The heated temperature of the debate speaks to its deep underlying emotional roots. It is senseless to try to solve the contentions in rational terms. A more productive venue seems to be to better understand its evolutionary moral origins. Haidt^3^ has proposed that beneath the political divide lie profound divergent emotional and moral dispositions.

Haidt's^3^ argument is three-tiered. First, moral judgments are aroused by powerful emotions, and are automatically processed. Rational moral justifications are provided after the fact. Second, cross-cultural studies disclose the emotional origins of half a dozen universal moral motifs. These moral motifs are evolutionarily stable adaptive strategies whose parameters are differentially set across cultures. Third, moral parameter setting serves to promote coalition formation, intragroup cooperation and intergroup competition.

In the following, the main components of the moral intuitionist interpretation of the political divide proposed by Haidt^3,10^ are discussed, hinting to its possible neural underpinnings.

**Moral intuitionism and its neural underpinnings.** Confronted with moral dilemmas disclosing violations of some deontological prescriptions without causing any kind of harm to third parties, strong emotional reactions arise.^11^ Individuals tend to vehemently condemn the character's behaviors. Asked to justify their judgments, participants encounter severe difficulties logically constructing their arguments.

Results of Haidt et al.^11^ and several other social psychological experiments lead to a moral intuitionist or dual process view of moral judgments.^3,10^ According to this view, moral judgment is initially based on automatically processed emotional arousal. It is only *post facto* that more controlled information processing is engaged in order to provide a logical justification of the moral stance.

CAS neuroscientific research is largely according to the moral intuitionist position. Observing the behavior of patients with ventromedial prefrontal cortical (vmPFC) lesions, Damasio^12^ famously proposed the somatic marker hypothesis. Individuals with lesions in the vmPFC, insula, amygdala and other related regions encounter difficulties anticipating the (moral) consequences of their behavior because they lack some kind of visceral/emotional input that is crucial to adaptive decision-making.

Later studies largely corroborated and expanded Damasio's^12^ argument. For example, Koenigs et al.^13^ presented moral dilemmas to focal brain damaged patients and asked for their judgments. The dilemmas had similarities to the ones used by Haidt et al..^11^ The vignettes were planned so as to promote strong emotional reactions without utilitarian violations of anybody's rights or well-being. Patients with vmPFC lesions had no difficulties endorsing utilitarian solutions that were strongly rejected by normal individuals and individuals with brain lesions elsewhere. In this sense, vmPFC patients may constitute the most perfect realization of the Anglo-Saxon utilitarian. These results have been corroborated by other studies involving acquired vmPFC lesions,^14,15^ frontotemporal dementia,^16^ and alexithymia.^17^

Both psychological and neuroscientific research endorse the intuitionist view of moral judgment, rooting it in deeper emotional processes. The forebrain reward and alerting systems and their cortical expansions seem to underlie these emotional influences on moral judgments.

According to the intuitionistic perspective, moral feelings are derived from more basic emotions such as love, joy, fear, anger and disgust.^3^ The moral underpinnings of these emotions are being increasingly recognized.^18^ The research program conducted by Haidt suggested that political affiliation may be related to temperament characteristics. Several models of temperament and motivated behavior are useful to understand the emotional motifs underlying political orientation.^19-21^

Temperament represents the reactive, affective-emotional foundations of personality.^18^ Factor-analytic studies led to the development of a trifactorial model of temperament, consisting of three independent but interacting dimensions of positive affect, negative affect and control. A systematic review of functional neuroimaging studies helped to identify the neural underpinnings of these dimensions.^21^ Positive affect involves a neural circuit with important knots in the left frontal cortex and nucleus accumbens. Negative affect is related to neural networks encompassing the right frontal cortex, amygdala and other structures. Finally, control or constraint is associated to error monitoring, detection and correction mechanisms with an important hub in the anterior cingulate cortex.

These results suggest a model of motivated behavior comprising approximation, avoidance and control.^19,20^ Approximation behaviors are dependent on dopaminergic circuits around the nucleus accumbens. Avoidance behaviors are associated to the amygdala. Two levels of control may be characterized, namely, the vmPFC adjudicates between approximation and avoidance, while the anterior cingulate mechanisms balance between emotional regulation strategies associated with the vmPFC and more cognitive strategies implemented around the dorsolateral PFC (dlPFC).

The interplay of emotions and cognitive processes in moral behavior and judgment has been contextualized in the dual process theory of information processing.^3,22^ According to this view, morally relevant situations promote emotions in ventral systems such as the amygdala and vmPFC, that are implicitly and automatically processed, whereas moral judgment relies on controlled cognitive processes mediated by the dlPFC. This has been famously investigated and reviewed by Greene^22^ through several functional neuroimaging studies with moral dilemmas. Utilitarian solutions promoting the lesser evil are associated with dlPFC activations whereas rejection of utilitarian solutions revolves around vmPFC activation. Where do these emotions come from, in the evolutionary sense?

**Cross-cultural set of moral motifs, personality and political partisanship.** Anthropological research has disclosed half a dozen moral motifs that are universally present in all human cultures.^23^ These moral motifs rest on emotional foundations and constitute epigenetic parameters that are differentially set across cultures and subcultures.

Emotions promoting socially regulated moral feelings and motifs are exemplified by fear, disgust, affiliation, cooperation and competition, etc. Haidt^3^ systematized the main universal motifs as:

1) Care versus harm;
2) Equality versus cheating;
3) Freedom versus oppression;
4) Loyalty versus treachery;
5) Authority versus subversion; and
6) Sanctity versus degradation.

The way these moral parameters are epigenetically set across different cultures and subcultures is illustrated by partisan differences.^3^ All parameters must be set, but relative weights vary as changing circumstances may place some moral values in conflict. Investigations of interindividual personality variability may be systematically associated with political positions endorsed.

Graham, Haidt and Nosek^5^ observed that politically conservative individuals tend to be more tolerant to inequality, less tolerant to change, more conscientious, and more disgustful towards cheating, sanctity, authority, and loyalty violations. Liberals, in turn, tend to be more open to experience and more empathic, valuing care and equality and abhorring harm and oppression. Conservatives are highly sensitive to cheating and to individuals receiving a larger share than they deserve. Liberals endorse the positive concept of liberty, being extremely sensitive to human rights violations. Equality and social justice are sanctified by liberals.

Libertarians constitute a tiny but growing group that has only more recently been investigated from a psychological standpoint.^6^ Libertarians strongly endorse liberty in the negative sense of freedom of coercion, relegating all other moral values to second place. They tend to be more rational and less emotional and affiliative. In this sense, libertarians have similarities to vmPFC utilitarians^13^ and individuals with Asperger's syndrome.^24^ They are predominantly male, highly intelligent and educated. It could be said that liberty as freedom from coercion is sanctified by libertarians.

Investigations on the neural underpinnings of political partisanship are incipient but growing. One study used fMRI to investigate the neural basis of political partisanship.^25^ Subjects were expected to endorse a series of statements that had previously been multidimensionally scaled to three dimensions. Endorsement of an individualist (versus collectivist) orientation was associated with activations of the medial prefrontal cortex and temporoparietal junction. Conservatism (versus liberalism) was associated with the dorsolateral prefrontal cortex. Finally, political radicalism (versus moderate positions) significantly activated the ventral striatum and posterior cingulate. The exact meaning of this individual variability in young American students observed by Zamboni et al.^25^ is not at all clear. But it is noteworthy that the regions implicated have been strongly associated with social cognition (default network and mind reading), working memory, executive functions and general intelligence, as well as the reward circuitry.

Further studies have explored group variability in the decision-making and reward circuitry of liberals and conservatives. Amodio et al.^26^ recorded larger error-related negativities associated with liberal vs. conservative positions. The authors suggested that the association between an anterior cingulate neurophysiological conflict-resolution marker and liberal positions may indicate sensitivity to cues for altering habitual response patterns. These data are consistent with volumetric analyses disclosing higher gray matter volumes of the anterior cingulate in liberals and of the right amygdala in conservatives.^27^

In a study by Schreiber et al.,^28^ liberals and conservatives did not differ in risk-taking behavior. However, different activation patterns were correlated to partisanship. Liberals activated the left insula more and conservatives the right amygdala.

Tentatively, it could be argued that personality characteristics of individuals with conservative political tendencies are derived from avoidance and constraint mechanisms, whereas liberal and libertarian positions center on themes of exploration of the environment and openness to experience.^5,6^

Another socioemotional dimension possibly related to morality and political partisanship is affiliative behavior.^29^ Affiliative behaviors are integrated at several distinct levels of the brain from oxytocinergic mechanisms at the diencephalon to more cognitive mechanisms related to theory of mind and empathy at the medial cortical default network,^30^ and mirror neuron system.^31^ Liberals were found to be consistently more empathic whereas libertarians lacked empathy. Conservatives were situated mid-way between these extremes in empathic traits.^5,6^

It is premature to interpret the scarce data on the neural underpinnings of political partisanship. However, the extant data are compatible with models relating personality traits to executive functioning.^32,33^ Brain activation patterns in liberals are consistent with greater cognitive flexibility and risk-taking behavior. Conservative brains may be wary of risk and more adherent to habitual and steady patterns of behavior.

If group variations in political partisanship are systematically associated with the neural substrate of moral judgments, it is necessary to examine the presumptive adaptive functions of this variability. This is the subject of the next section.

**Evolutionary adaptive functions of political partisanship.** One major aspect of human evolution is the cognitive demands imposed by social life in increasingly larger and complex groups. Current models suggest that the cognitive pressures imposed by social cooperation and competition constitute an important booster of human evolution.^34^

From the evolutionary standpoint, increasing group size is associated with increased brain volume and cognitive capacity.^35^ Life in increasingly complex groups may be related to the evolution of language and mind reading serving to promote coalition formation. Coalition formation is a universal characteristic of human cultures that imposes several cognitive demands required for intragroup cooperation and intergroup competition.

Sociocognitive abilities operate at the level of group selection and impose an arms race dynamic on human cognitive evolution. In order to outcompete rival groups, increased complexity of intragroup cooperation and intergroup antagonistic strategies are required. Political partisanship reflects the emotional and ideological cement of intragroup and intergroup evolutionary dynamics.

One interesting model for studying the role of coalition formation and competition in human evolution is ethnic prejudice.^36^ Fear seems to be the dominant emotion underlying xenophobia and ethnic prejudice. Ethnic-related fear may be acquired by direct Pavlovian conditioning, vicarious observation or verbal persuasion.^37,38^ Learning of ethnic-related fear activates the amygdala (the left amygdala in the case of verbal persuasion).

Ethnic prejudice may manifest both explicitly and implicitly. One procedure to detect implicit ethnic prejudice is the implicit association test (IAT^39^). In the racial IAT, participants initially learn to associate one response key to positive characteristics of their own ethnic group and a second response key to negative characteristics of another ethnic group.^40^ In a second phase of the experiment, participants are required to invert the contingencies. In this second phase, response keys used for own race and positive characteristics should be used for own race and negative characteristics, while the second key, previously used to associate another ethnic group to pejorative characteristics, must be used to connect this ethnic group to favorable traits.

Difficulties in learning the contingency during the second phase of the racial IAT are interpreted as manifestations of implicit ethnic prejudice.^40^ Reaction times increase as the individual must resort to controlled processing to inhibit prejudiced race associations. This interpretation is endorsed by fMRI studies showing early amygdala activation, possibly related to automatic processing, followed by later, controlled processing in the prefrontal cortex on the racial IAT.^41^

Implicit and explicit prejudice is developmentally dissociable.^42^ Implicit ethnic prejudice in white Americans remains relatively constant throughout development from childhood to adult age. At the same time, explicit endorsement of ethnic prejudiced statements reduces significantly.

The connection between ethnic prejudice and coalition formation was suggested in a psychological experimental study by Kurzban, Tooby and Cosmides.^43^ In a basketball supporters' context, Kurzban and colleagues were able to substitute team-related antagonism for ethnic-related antagonism.

The study by Kurzban et al.^43^ not only suggests a direct connection of ethnic prejudice with intragroup-extragroup dynamics, but also indicates that the criteria for coalition formation and maintenance may be relatively flexible and subject to cognitive manipulations.

Further work by Richeson et al.^44-46^ suggests that ethnic prejudice may be voluntarily suppressed at the expense of mental effort. This is good news, as it suggests that parameters of coalition formation are amenable to behavioral and cognitive interventions. Conscious effort to suppress coalitional partisanship and to cooperate constructively with differently-minded individuals would not be an insoluble problem if individuals always acted in a bona fide manner.

Individuals tend to endorse their political views in fanatical ways, suggesting that self-deception is an important component of partisanship. In the words of Haidt,^3^ political ideology "binds and blinds". It serves to cohere individuals in groups, but at the same time makes them neglectful of ideological and group-serving manipulations.

**Self-deception serving to promote political partisanship.** Self-deception is a cornerstone in the evolution of morality.^47,48^ Cooperative intragroup behavior is based on reciprocal altruism. Communities that evolved cooperation based on reciprocal altruism are prey to the free rider problem.^49^ Any non-reciprocating free rider who entered a community of reciprocal altruists would easily propagate their non-cooperative genes to the next generations. And the fundamentals of the group cooperation would be undermined.

The evolution of cheating detection mechanisms is one strategy that evolved in the arms race of coalition formation. Cooperating individuals are endowed with nonconscious red flag signals or somatic markers that guide the decision-making process and reduce the risk of being predated by cheaters.^49^ Disgust toward cheaters and advantage takers is one of the main traits of the conservative mind.^3,50^

The arms race of coalition formation leads then to the evolution of self-deception as an important strategy to counterbalance cheat detection. If one is to lie, the more effective the lie will be if the liar believes in it. Good liars believe in their own lies. This could represent the evolutionary origins of unconscious mental processes.^47,48^

Wishful thinking is one dimension of self-deception. Self-deception and moralism may be an effective strategy to cheat others. But it also has collateral effects. The self-cheater may be prey to other self-deceivers. This seems particularly to be the case of confidence tricks or get-rich quick schemes.

A conman makes an almost irrefutable proposal. The proposal is too good to be true. But it is irresistible for individuals with a predisposition to take advantage of others. In trying to deceive others, the individuals are cheated themselves. Patients with vmPFC lesions are especially prone to these confidence tricks.^12^

Confidence tricks and self-deception help to understand the relationships of populist politicians and their needy constituency. Many populist leaders invest themselves with picaresque traits. They play the smart, simple guy who came from the bottom, but who is able to outplay the wealthier and more educated. Macunaíma, the hero with no character in the homonymous novel by Mário de Andrade,^51^ is one such example from contemporary Brazilian literature.

Some political leaders are fleshing out this character. They claim they came from the bottom, deny being educated, transform ignorance into virtue, and at the same time play the sage, portraying the guy who knows things and is able to transform reality. People trust these characters because they are in need. However, they are also trusting because they believe in get-rich quick schemes. They believe it to be possible to access wealth without hard work or savings.

The liberal narrative tells of greedy capitalists who refuse to share their wealth with the less fortunate.^2^ This narrative assumes that the economy is a zero-sum game, and that resources should be distributed as if collected from a pot, without regard for effort. Liberals are very sensitive to the suffering caused by misery, but they are unable to foresee the consequences of lack of proportional reward for effort and the role that reward plays in wealth production.

The conservative and libertarian narratives assume that there is no free lunch,^9^ and that the welfare state also has its collateral effects. One important collateral damage of the welfare state is the reward of dependency, the emergence of an underclass of individuals who through generations are unable to autonomously earn their livelihood.^50^ Political clientelism is a mutual reinforcement relationship between populist leaders and the dispossessed masses. The masses vote for and depend on the leader's largesse.

The neural bases of self-deceptive behavior remain largely unknown. Functional neuroimaging studies of lying and other forms of hetero-deceptive behaviors have disclosed widely distributed patterns of activation in areas associated with executive functions, the default network and mind reading.^52^

No convincing evidence on the functional neuroimaging basis of self-deception has been found. This may rest on the fact that functional neuroimaging studies rely on a stimulus-response paradigm, and it is virtually impossible to instruct somebody to self-delude.

Notwithstanding these difficulties, some conjectures can be put forward. Self-deceptive behavior is frequently observed after neurological damage. Neuropsychological patients with lesions in the vmPFC and basal forebrain regions are prone to self-deception behaviors such as susceptibility to confidence tricks^12^ and confabulation.^53^ The several forms of anosognosia may also be taken as manifestations of self-deception and are correlated to fronto-parietal lateral cortical lesions.^54^ Alexithymia is a psychiatric symptom consisting of difficulties reading or interpreting one's own feelings (affective agnosia) and has been linked to dysfunctions in the default and mind reading networks.^55^

We are not suggesting that the garden variety of self-deception observed in daily life or politics is associated with any kind of brain damage. However, neuropsychological evidence indicates the possible neural substrate of self-deception. It is possible then, that interindividual variation in the neural substrate of personality, motivation and moral feelings could predispose some individuals to self-deception. And this would also manifest in political partisanship.

## DISCUSSION

Our excursion through the moral-psychological and neuroevolutionary underpinnings of political partisanship served as a case-example to examine possible contributions of CAS neuroscience to social development. Following Haidt,^3^ research has contributed greatly toward elucidating possible mechanisms responsible for partisan behavior and its relative imperviousness to change:

1) Political partisan behavior rests upon moral emotions. Early steps in moral judgment automatically evoke deep emotions, and it is only later on that controlled processing takes over to generate rationalizations of the emotionally-laden choices;
2) The moral foundations of partisan behavior consist of a series of universal moral values whose parameters are epigenetically set across different cultures and subcultures;
3) Different political parties assign different weights to a set of universal moral values that may contradict themselves depending on the circumstances;
4) Liberals, for example, endorse a positive view of liberty as a human right while libertarians and conservatives value a negative concept of liberty as freedom from coercion;
5) Liberals are empathic and extremely sensitive to harm, inequality and oppression. Conservatives are more sensitive to cheating, authority, loyalty and sanctity violations. Libertarians tend to behave in a more rational or utilitarian way, considering future consequences of choices and being less sensitive to emotional appeal;
6) Self-deception is an important adjunct to political partisanship, explaining why partisanship "binds and blinds", why it is so impervious to change, and, at the same time, predisposes individuals to confidence tricks;
7) Emotional and ideological partisan mechanisms represent an evolutionary stable adaptive strategy, selected to promote intragroup cooperation and intergroup competition in the context of the formation of a coalition and maintenance of an arms race;
8) Interindividual variability in political affiliations may be systematically associated with morphological and functional diversity in brain structures involved with personality, arousal, reward and motivation, executive functions, default state and mind reading;
9) Neuroscientific results are compatible with a dual process model of political affiliation, consisting of early and automatic emotionally arousing events recruiting the amygdala and later, more controlled processes centering on self-regulatory mechanisms;
10) Under some circumstances, behavioral and cognitive interventions may enjoy limited success in changing some of the undesirable consequences of political partisanship and promoting more cooperative forms of behavior between differently-minded people.

Cognitive and neuroscientific research is increasingly helping to elucidate the psychological and neuroevolutionary mechanisms underlying political partisanship. Yet, so far it has not taught us how to reduce the antagonistic effects of partisanship and how to effectively promote cooperation between politically differently-minded individuals. Other questions that remain open are:

1) Could neuroscientific evidence be used to settle political differences?;
2) Could neuroscientific evidence be used to rationally plan social improvement as desired by liberals or libertarians, or should we guard against this possibility as proposed by conservatives?;
3) Which kind of translational research is required to effectively use neuroscientific evidence, if so decided, in the betterment of social affairs?;
4) Will neuroscience ever produce a paradigm shift in the study of the cognitive, affective and social bases of human behavior, or will it remain a strategy for testing hypothesis generated elsewhere?

The first question seems to be an aporia. The other three questions are an empirical matter.
